# Correlation between Internet addiction, depression, anxiety and stress among undergraduate medical students in Azad Kashmir

**DOI:** 10.12669/pjms.35.2.169

**Published:** 2019

**Authors:** Arslaan Javaeed, Maria Bint Zafar, Madiha Iqbal, Sanniya Khan Ghauri

**Affiliations:** 1 *Arslaan Javaeed, MBBS, M.Phil. Poonch Medical College, Rawalakot, Azad Kashmir, Pakistan*; 2 *Maria Bint Zafar, MBBS*; 3 *Madiha Iqbal, MBBS. Poonch Medical College, Rawalakot, Azad Kashmir, Pakistan*; 4 *Sanniya Khan Ghauri, MBBS, MRCEM. Department of Emergency Medicine, Shifa International Hospital, Islamabad, Pakistan*

**Keywords:** Internet addiction, medical students, DASS21, anxiety, depression, stress

## Abstract

**Objective:**

To find out the correlation between internet addiction and depression, anxiety, and stress among undergraduate medical students in Azad Kashmir.

**Methods:**

A cross-sectional study including 210 undergraduate medical students (first to the fifth year) was done in Poonch Medical College, Azad Kashmir. The data collection tools were DASS21 questionnaire and Young’s internet addiction questionnaire. Spearman rank correlation test was done to see the correlation between internet addiction and depression, anxiety, and stress. Data were analyzed by SPSS v23 at a 95% confidence interval.

**Results:**

A very high prevalence (52.4%) of moderate to extremely severe internet addiction was observed among the respondents. The mild positive correlation between internet addiction and depression was identified (p <.001) and similar type of correlation was observed between internet addiction and stress (p .003). However, anxiety and internet addiction were not significantly correlated. The prevalence of anxiety and depression among the males were higher than the females, whilst the stress level was almost the same across gender.

**Conclusion:**

Internet addiction has been found to be associated with various psychiatric diseases. In this study, we also observed such correlation. We have also observed a very high level of internet addiction among medical students. The prevalence of internet addiction may further increase in the coming years as the internet will become more cheap, available and include more high quality psychologically addictive contents.

## INTRODUCTION

Over the last fifteen years, the number of internet users increased by one thousand fold. The addictive internet usage also proliferated significantly in the last decade. And it has been found to be associated with younger age internet users.^1^ Although internet addiction (IA) is not included in the Diagnostic and Statistical Manual of Mental Health Disorder, Fifth Edition, its association was identified with various psychiatric conditions.^2^ People who spend more than 38 hours a week online, are considered to have an Internet addiction. Internet addiction does not involve the use of an intoxicating drug, but it is very similar to pathological gambling in terms of brain biochemistry.^3^ Internet addicts tend to become less responsible for their actions and suffer from social isolation.^4^

Internet is an essential part of life especially for the medical students due to its need for education, research, social networking, and information sharing, banking, shopping etc.^5^,^6^ Medical students fall in the vulnerable group due to their age, need for internet use and stressful medical training.^7^,^8^ A comorbid psychopathology systematic review in excessive internet use revealed that 75% of the examined studies reported a significant correlation between pathological internet use and depression.^9^ A previous study showed the significant relationship between IA and psychiatric symptoms such as depression, obsessive compulsion, interpersonal sensitivity, anxiety, hostility, phobic anxiety, paranoid ideation, and psychoticism. With longer use, more psychiatric symptoms occur.^10^ This study was aimed to assess the correlation between internet addiction and depression, anxiety, and stress among the medical students of Azad Kashmir.

## METHODS

The cross-sectional study was conducted at Poonch Medical College, Azad Kashmir including 210 undergraduate medical students. The medical students were from first to fifth years. Study duration was from April to October 2018. The data were collected using interviewer-administered, well-known DASS21 questionnaire and Young’s internet addiction questionnaire. The DASS21 questionnaire has been successfully used across the world in many previous studies to measure depression, anxiety, and stress.^11^-^13^ The DASS21 questionnaire previously showed a high level of internal consistency (Cronbach’s alpha = 0.70 and above).^14^ The DASS21 questionnaire contains 21 4-points Likert scale questions, of which 7 for measuring depression, seven for anxiety, and the remaining seven questions for identifying stress. The Young’s internet addiction questionnaire includes twenty Likert scale questions, each of which measures internet addiction at 0 to 6 points scale.^15^ After the extensive literature search, the prevalence of internet addiction was observed to be from 2% to 7.9%.^16^-^18^ Due to variation of prevalence rate in the literature we have assumed a high prevalence rate of 15% to make sure the sample size was adequate. By applying the popular formulae N = Z^2^* P(1-P)/e^2^, we have obtained a sample size of 196. List of all medical students was collected from the database of the medical college. From there 250 medical students were randomly selected using a random number generator of SPSS v23. The interviewer himself approached all 250 medical students with the DASS21 questionnaire. Total 210 medical students gave the consent and completed the questionnaire (Response rate of 84%). These 210 medical students were included in the study. Ethical approval was obtained from the ethical review board of Poonch Medical College.

Descriptive statistics were used to present the data in tables and charts. Cronbach’s alpha test was done to check the internal consistency of DASS21 and Young’s IA questionnaires. Spearman rank correlation test was done to correlate the level of internet addiction and depression, anxiety, and stress levels. The analysis was performed in 95% confidence interval using the Statistical Package for Social Science (SPSS), version 23.0 (IBM, Armonk, NY, USA).

## RESULTS

Among the 210 respondents, 75 (35.7%) were male. Baseline characteristics are presented in Table-I. The Chronbach’s alpha value of DASS21 questionnaire and Young’s IA questionnaire for the current study was 0.820 and 0.852. According to DASS21 score calculation, extremely severe depression, anxiety, and stress were observed among 40 (19.0%), 97 (46.2%) and 5 (2.4%) medical students respectively. (Table-II) Only 7 (3.3%) medical students did not have any internet addiction. The prevalence of moderate to severe internet addiction among the medical students was 52.4%. (Chart 1). All three of severe internet addicts were females. Extremely severe depression was higher among the male respondents 21 (28%) compared to females 19 (14.1%). Anxiety was also observed more among the males 69 (92.0%) than females 109 (80.7%). Moderate to severe internet addiction was observed almost equally in males and females, around 36%.

**Table-I:** Baseline characteristics of the respondents.

Characteristics	N (%)
Age in years (mean ± SD)	21.84 ± 1.78
***Gender***	
Male	75 (35.7)
Female	135 (64.3)
***Year of study***	
First year	36 (17.1)
Second year	43 (20.5)
Third year	38 (18.1)
Fourth year	46 (21.9)
Fifth year	47 (22.4)

**Table-II:** Depression, anxiety and stress levels of the respondents according to DASS21 scores.

Category	Depression N (%)	Anxiety N (%)	Stress N (%)
Normal	66 (31.4)	32 (15.2)	88 (41.9)
Mild	25 (11.9)	4 (1.9)	47 (22.4)
Moderate	42 (20.0)	45 (21.4)	33 (15.7)
Severe	37 (17.6)	32 (15.2)	37 (17.6)
Extremely severe	40 (19.0)	97 (46.2)	5 (2.4)

There was statistically significant mild uphill correlation between depression and internet addiction (correlation coefficient .329 and p <.001), and an even milder uphill correlation between stress and internet addiction (correlation coefficient .202, p .003). However, anxiety was not significantly correlated with internet addiction (p .391). (Table-III).

**Table-III:** Correlation between internet addiction and depression, anxiety, and stress.

		Depression	Anxiety	Stress
Internet addiction	Correlation coefficient	0.329	0.060	0.202
	p-value	<0.001	0.391	0.003

## DISCUSSION

Psychological stress on the medical students is relatively higher than other disciplines. A multitude of factors may be responsible for these high stress level.^19^ It is very difficult to know what percentage of the stress comes from internet addiction alone. The global prevalence of internet addiction was 6.0%.^17^ In contrast, we have found 96.3% of the medical students to have some degree (mild to extremely severe) of internet addiction. A study done in Saudi Arabia revealed a very high level of depression, anxiety, and stress among the medical students. But the stress level may change depending on the propinquity of examination dates.^20^

An Iranian study showed a higher prevalence of IA among the females which goes against the current study findings as here we have observed a similar level of internet addiction across the gender.^21^ The significant difference in the prevalence of IA and its relationship with depression, anxiety, and stress were observed in the literature. This might be because of IA is not constant in all time. The internet is becoming more cheap, available and including more services as time progresses. A study done on college students in India revealed a positive correlation between IA and depression, anxiety, and stress.^22^ However, in this study, we did not find any significant correlation between IA and anxiety although the prevalence of moderate to extremely severe anxiety was very high, 82.8%.

**Fig.1:**
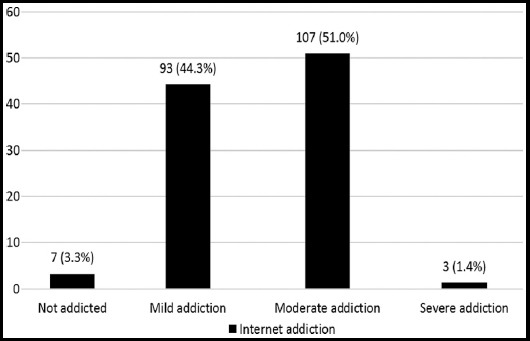
Frequencies and percentages of respondents with different degree of internet addiction.

## CONCLUSION

Internet addiction has already been associated with a multitude of psychiatric disorders. We have observed a significant relationship between internet addiction and anxiety and stress. The prevalence of internet addiction and its association with psychosocial problems has the potential to increase in future as the availability of internet in general and enrichment of the virtual world with more high-quality, attractive and addictive digital contents are rising.

### Limitations of the Study

Being a single centered study, it may not include the representative samples which make the generalization of the study finding appalling. The psychological stress level of the medical student may be subject to change depending on many other factors. The study did not involve any confounders.
